# Feasibility and safety study of a new device (Odón device) for assisted vaginal deliveries: study protocol

**DOI:** 10.1186/1742-4755-10-33

**Published:** 2013-07-02

**Authors:** The World Health Organization Odon Device Research Group

**Affiliations:** 1 World Health Organization

**Keywords:** Assisted vaginal delivery, Obstructed labour, Intrapartum complications, Odón device, Maternal mortality, Maternal morbidity, Fetal mortality

## Abstract

**Background:**

Intrapartum complications are responsible for approximately half of all maternal deaths, and two million stillbirth and neonatal deaths per year. Prolonged second stage of labour is associated with potentially fatal maternal complications such as haemorrhage and infection and it is a major cause of stillbirth and newborn morbidity and mortality. Currently, the three main options for managing prolonged second stage of labour are forceps, vacuum extractor and caesarean section. All three clinical practices require relatively expensive equipment (e.g., a surgical theatre for caesarean section) and/or highly trained staff which are often not available in low resource settings. The specific aim of the proposed study is to test the safety and feasibility of a new device (Odón device) to effectively deliver the fetus during prolonged second stage of labour. The Odón device is a low-cost technological innovation to facilitate operative vaginal delivery and designed to minimize trauma to the mother and baby. These features combined make it a potentially revolutionary development in obstetrics, particularly for improving intrapartum care and reducing maternal and perinatal morbidity and mortality in low resource settings.

**Methods/design:**

This will be a hospital-based, multicenter prospective phase 1 cohort study with no control group. Delivery with the Odón device will be attempted under normal labour and non-emergency conditions on all the women enrolled in the study. One-hundred and thirty pregnant women will be recruited in tertiary care facilities in Argentina. Safety will be assessed by examining maternal and infant outcomes until discharge. Feasibility will be evaluated by observing successful expulsion of the fetal head after one-time application of the device under standardized conditions (full cervical dilation, anterior presentation, +2 station, normal fetal heart rate).

**Trial registration:**

Australian New Zealand Clinical Trials Registry (ANZCTR). Identifier: ACTRN12613000141741

## Background

Intrapartum complications are responsible for approximately half of all maternal deaths, and two million stillbirth and neonatal deaths per year [1-5]. Prolonged second stage of labour is associated with potentially fatal maternal complications such as haemorrhage and infection and it is a major cause of stillbirth and newborn morbidity and mortality. It also increases the risk of vaginal and perineal tears and instrumental deliveries. The three main options for managing prolonged second stage of labour are forceps, vacuum extractor, and caesarean section. The use of forceps and vacuum extractor during prolonged second stage of labour is associated with increased perinatal morbidity [6-15]. Cephalohematoma and retinal haemorrhages have been associated with the use of vacuum extractor while external ocular injury and facial nerve palsy have been associated with forceps deliveries [15]. Evidence indicates that vacuum extraction is significantly less likely to achieve successful vaginal delivery than forceps but it is associated with lower caesarean deliveries, probably because forceps are sometimes used after failed vacuum extraction. Vacuum extraction is also contra-indicated in women who are HIV positive or with unknown status. On the other hand, use of vacuum extractor results in lower rates of perineal trauma, tears, requirements for pain relief and incontinence in comparison with forceps [15].

Caesarean delivery in the second stage of labour accounts for approximately one fourth of all primary caesarean sections [16]. Although second stage caesarean sections are sometimes appropriate for medically indicated reasons, many of them could be avoided by obstetricians skilled in performing operative vaginal deliveries [17]. However, fewer health care professionals are receiving appropriate training in operative vaginal deliveries. This change in medical curriculum is contributing to the observed declining trend in the use of operative vaginal deliveries and presents a challenge for maintaining and developing these skills [18].

### Rationale for the trial: opportunity for development of alternatives

All three available clinical practices for managing prolonged second stage of labour require relatively expensive equipment (e.g., a surgical theatre for caesarean section) and/or highly trained staff that are often not available in low resource settings.

Evidence indicates that the majority of intrapartum deaths in low-resource settings could be prevented through the introduction of innovative strategies that would increase accessibility of obstetric care (e.g., task shifting) [4]. Technological developments such as the Odón device that reduces the skill level and equipment required to provide quality obstetric care are clearly needed.

**Figure 1:**
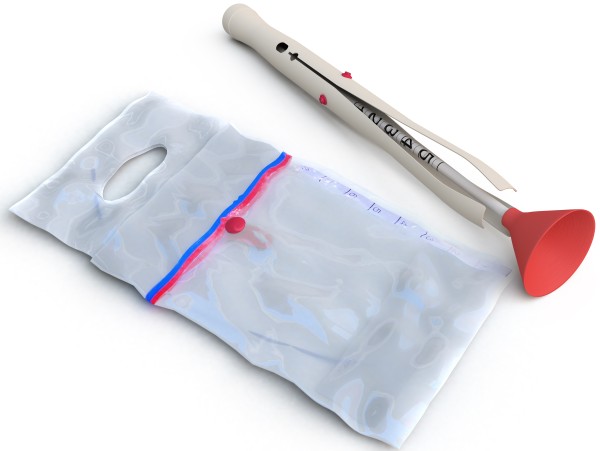
The Odón device: a new, simple instrument for assisted vaginal deliveries.

The Odón device (see Figure 1; http://www.odondevice.com/) is envisioned to be a possible alternative to available management options for complicated second stage of labour. It may be potentially safer and easier to apply than forceps/vacuum extractor for assisted deliveries, and a safe alternative to some caesarean sections. It has potential for wide application in low-resource settings, even by mid-level providers. If proven safe and effective, the Odón device will be the first innovation in operative vaginal delivery since the independent development of forceps and the vacuum extractor centuries ago. The Odon device could play a major role in improving intrapartum obstetric care in low resource settings by making possible effective management of intrapartum complications in facilities lacking surgical capacity and/or personnel adequately trained in the use of forceps and the vacuum extractor. Future research may explore additional uses of the device to prevent vertical transmission of infections during childbirth.

### Description of the device and preliminary result in simulators

**Figure 2:**
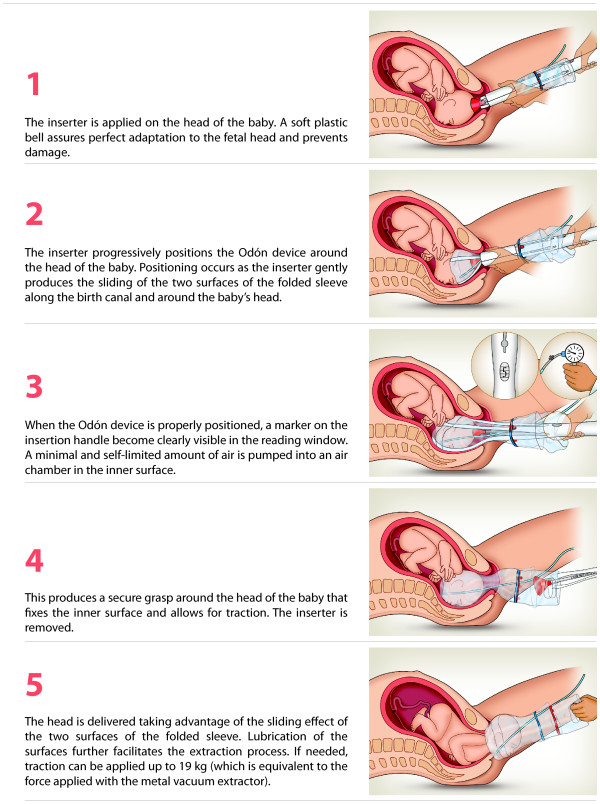
Visualization of the use of the Odón device.

In comparison with available instruments for operative vaginal delivery (forceps and vacuum extractor), the Odón device presents specific features that could potentially reduce the risk of maternal and fetal injury. The Odón device is made of inexpensive film-like polyethylene material (Figure 1) and it is designed to be easily applied with the help of an inserter requiring minimal training. The application and use of the Odón device is shown in Figure 2.

Studies in animals have been ruled out for several reasons. In most animals, the head is not the presenting part in normal delivery. Theoretically, experimentation of the Odón device would have been possible in superior primates (chimpanzee, gorilla, and orangutan). However, in practice this option is not feasible because any experimental procedure concerning childbirth with these primates would require general anaesthesia, which would interfere with the physiological mechanisms of labour.

The Odón device was tested under conditions simulating normal delivery at the Simulation Centre of Des Moines University, USA in December 2008. The simulator S 575 (Noelle) which closely simulates a mother-fetus dyad in labour and delivery was used. Under those experimental conditions, the application of the Odón device consistently allowed for the immediate expulsion of the fetus in multiple trials. At that time, the device had never been tested in humans or animal models.

### Study hypothesis and objectives

The main objective of this study is to evaluate the safety and feasibility, in terms of ease of application and successful use of the Odón device in assisting vaginal delivery in singleton term pregnancies during the second stage of labour. The hypothesis is that the Odón device is safe, easy to use, allows for successful delivery, and is accepted by both women and obstetricians.

Safety will be assessed by examining maternal and infant outcomes. Feasibility will be evaluated by observing successful expulsion of the fetal head after one-time application of the device under standardized conditions (full cervical dilation, anterior presentation, +2 station, normal fetal heart rate) in singleton non-complicated pregnancies. Successful expulsion would be defined as the complete expulsion of the head of the baby with the device. Cases of crowning will be recorded. Successful application of the device will be defined as (1) reaching number 4 or 5 in the reading window of the inserter, (2) successful inflation of the device without leaks after the expulsion, and (3) successful expulsion.

### Study conceptual framework

This proposed protocol is embedded in a larger two-phased study:

PHASE 1: the device will be tested for safety and feasibility in terms of ease of application and successful delivery under non-emergency/normal delivery conditions. 130 women will be recruited for delivery using the Odón device. There will not be a control group. This proposed protocol refers to Phase 1 of this study.

PHASE 2: the device will be tested for preliminary efficacy (successful delivery of the baby without the need to resort to caesarean section/forceps/vacuum extractor) in deliveries with prolonged second stage of labour with an indication for not immediate operative delivery (allowing enough time for the application of the Odón device prior to standard of care) and no signs of fetal distress. 128 women will be randomly allocated either to: (1) standard of care (forceps/vacuum extractor); or (2) application of the Odón device. Preliminary efficacy will be assessed by determining the number of successful deliveries by Odón device.

## Methods/design

This will be a hospital-based, multicentre prospective cohort with no control group. Delivery with the Odón device will be attempted under non-emergency/normal delivery conditions on all women enrolled in the study.

### Outcome measures

The different measures collected will be used to assess safety and feasibility. Safety will be assessed by examining maternal and infant outcomes at labour, delivery and immediate postpartum care until discharge. Feasibility will be evaluated by observing successful expulsion of the fetal head after one-time application of the device under standardized conditions as explained above. The list of outcome indicators for Phase 1 are presented in Table 1.

**Table 1:** Study outcomes

**Labour and delivery**	
**At the time of device application**	• Time from application of device to delivery of the cephalic pole
	• Failure rate of device application (only one attempt will be made)
	• Incidence of:
	○ Operative delivery with forceps or vacuum
	○ Caesarean section
	We anticipate that there may be some cases in which the device will present the following: it cannot be applied, it is difficult to apply, it slips off, or it is ineffective. To determine the clinical circumstances under which such problems occur, the following clinical data will be collected:
	• Position of head
	• Station of head
	• Degree of caput succedaneum and moulding
**During and after delivery**	• Time taken to apply the device (in seconds; from the start to the removal of the inserter)
	• Successful application and inflation
	• Any slipping of the device
	• Number of contractions after application
	• Integrity of the air chamber after expulsion
	• Any other failure of the mechanical failure of the device
**Immediate postpartum period until discharge (24–48 hours)**	
**Maternal**	Incidence of:
	• Episiotomy
	• Lacerations of the low genital tract (vulvar, vaginal or cervical trauma)
	• Perineal lacerations including cervical laceration
	• Third or fourth degree perineal tear
	• Uterine rupture
	• Pelvic hematoma
	• Perineal or vaginal hematoma
	• Postpartum haemorrhage as routinely monitored at the centers
	• Postpartum endometritis or infection
	• Maternal blood transfusion
	• Maternal pain during insertion of the device
	• Maternal satisfaction
**Newborn**	Incidence of:
	• Respiratory rate at 2 hours
	• Birth trauma or injuries (skull fracture, caput succedaneum, cephalohematoma, cutaneous facial lesions and facial palsy, scalp, extracranial haemorrhages, subgaleal haemorrhage, subaponeurotic haemorrhage, intracranial injuries)
	• Low Apgar Scores (less than 7 at 1st minute and less than 7 at 5th minute)
	• Admission to Neonatal Intensive Care Unit
	• Length of stay (> 7 days) in Neonatal Intensive Care Unit
	• Retinal haemorrhage
	• Anaemia
	• Jaundice
	• Evidence of infection by local standards
	• Phototherapy
	• Fetal or neonatal death

### Study population

Pregnant women must meet all of the following inclusion criteria to be eligible for enrolment in the study and application of the Odón device during delivery:

1. Written informed consent obtained during an antenatal care visit, and always before the initiation of labour;

2. Age greater than or equal to 18 years and less than or equal to 35 years;

3. Singleton pregnancy;

4. Gestational age equal to or more than 37 weeks by best estimate according to local protocols;

5. Live fetus by the methods used with local protocols;

6. Fully dilated cervix;

7. Ruptured membranes (spontaneous or artificial);

8. Any anterior occiput presentation;

9. Station level equivalent to 2 cm or more below the spines (e.g. station +2 or lower);

10. Fetal heart rate pattern recorded by continuous fetal electronic monitoring meeting the following criteria:

a. Stable baseline rate between 110 and 160 beats/min

a. normal short and long term variability

a. absence of late decelerations.

### Exclusion criteria

Women presenting any of the following conditions will not be included in the study and the device will not be applied:

1. Suspected or confirmed maternal infection: suspected infection will be diagnosed if any of the following are present:

a) Fever greater than or equal to 38°C

a) Foul smelling amniotic fluid

2. Prolonged rupture of membranes (more than 24 hours)

3. Meconium stained

4. History of coagulation disorder

5. Previous uterine surgery

6. Intrapartum haemorrhage

7. Suspected cephalopelvic disproportion or need of operative delivery

8. Known recto-vaginal Streptoccocus B Haemolyticcus positive culture

9. Known HIV positive status

10. Any condition or circumstance that, in investigator’s opinion, would compromise the safety of the mother or the fetus.

### Sample size calculation

One-hundred and thirty pregnant women will be enrolled in phase 1 at participating centers. The sample size was determined in consideration of the study objective of assessing maternal and fetal safety. This sample size is considered feasible in the proposed research settings and will allow for detecting frequencies of maternal and/or fetal complications between 2% and 20% with a 95% confidence interval width not larger than 15%, as shown in Table 2.

**Table 2:** Estimated incidence rates of outcomes

**Outcome incidence rate (%)**	**Interval width**	**95% CI**
2.0	5.8	0.4 – 6.1
4.0	7.6	1.3 – 9.0
6.0	9.0	2.6 – 11.6
8.0	10.1	4.0 – 14.1
10.0	11.1	5.4 – 16.5
12.0	11.9	7.0 – 18.9
14.0	12.6	8.5 – 21.2
16.0	13.3	10.2 – 23.5
18.0	13.9	11.8 – 25.7
20.0	14.4	13.5 – 27.9

### Participant recruitment plan

At all study sites, women will be approached during antenatal visits by a midwife who will explain the study objective and procedures. If the woman agrees to receive more information about the study, she will be fully briefed by a member of the study team who will review the consent form and show her the Odón device and how it functions. An explanatory video will also be made available. The woman will not be asked to sign the consent form immediately. Instead she will be given the information sheet to take home to think about it and discuss with family members if she wishes. She can sign the consent form when she returns for the next antenatal visit. Consent will be obtained before the initiation of labour and women will be able to withdraw from the study at any time.

In this study all deliveries using the Odón device will be performed by an obstetrician. All study participants will have access to comprehensive emergency obstetric and newborn care throughout the project period.

The following multi-phase plan of recruitment and interim analysis will be put in place:

#### 1st recruitment period (completed)

This period included 5 cases for a preliminary evaluation of practicality, feasibility and safety. In addition to the above-mentioned inclusion and eligibility criteria, women had to be multiparous and to have previously experienced a successful vaginal delivery. Results were evaluated by the Data and Safety Monitoring Board (DSMB) that allowed for continuation of recruitment.

Participants in the 1st phase were asked for written consent to tape the application of the Odón device. These tapes were made available to the DSMB for safety evaluation purposes. Videos are kept confidentially only at the participating centers and at the WHO coordinating unit.

#### 2nd recruitment period (completed)

This period included an additional 25 participants (up to the 30th participant). Participants were all multiparous who had successfully delivered vaginally. Results were evaluated by the DSMB, which allowed for continuation of recruitment.

#### 3rd recruitment period

This period will include multiparous and nulliparous women. The stepwise approach will be continued until the target of 130 cases is reached. The DSMB will review the study results after the application of the device in the first 5–6 nulliparous women (for each site/country) and, if no significant problems are detected, continue recruitment. Afterwards, the DSMB will meet regularly after every 15 new deliveries in order to review that information and discuss any outcome.

### Data collection, data management and analysis

Case Report Forms (CRF) specifically designed in the WHO Coordinating Unit at UNDP/UNFPA/UNICEF/WHO/World Bank Special Programme of Research, Development and Research Training in Human Reproduction (HRP) for this study will be used for data collection. Data will be collected prospectively by the researchers at the study centres and data checking and entry of the data collection forms will be completed as much as possible at the local centres. Initial data collection will be on paper forms and then data will be entered onto a web-based online open-source data entry system (Open Clinica) developed and coordinated by the trial coordinating unit at HRP. The online data entry system minimizes the delays in data queries and completion of forms. These procedures have been used in previous multicentre trials and proven to be efficient and compliant with the HRP/WHO Standard Operating Procedures and Good Clinical Practice (GCP) guidelines. Similarly, HRP has good experience with online data entry systems from several international multicentre studies conducted in the past five years. Descriptive analysis will be carried out to show the frequencies of maternal and fetal complications, which will be reported as rates or means and 95% confidence intervals.

### Main problems anticipated and proposed solutions

The major anticipated problem is achieving adequate patient recruitment. Current experience at CEMIC University Hospital in Buenos Aires, Argentina and the high number of deliveries attending on an annual basis at the proposed participating centers indicates favourable conditions for study implementation and the likelihood that patient recruitment will be successful. In addition, the same obstetricians will assist the patient during the prenatal, delivery, and postpartum periods. This consistency in service provision will promote a close physician/patient relationship, which will allow for a comprehensive explanation of the objectives, benefits and risks related to the study to women and their partners. The physician will have enough time to familiarize women with the procedures prior to the onset of labour. Participating study sites will provide all facilities and resources for study implementation.

### Applicability of results

The expected clinical outcome of the proposed study is that the Odón device will, when applied during the second stage of labour, successfully and safely facilitate vaginal delivery.

These results will also inform the development of future research conducted through large pragmatic randomized clinical trials to compare safety and effectiveness of the Odon device with caesarean section, forceps and vacuum extractor in cases of prolonged second stage of labour with indicated operative delivery for maternal and/or fetal complication.

Should this method prove to be effective and safe, it could contribute to a reduction in maternal and newborn complications related to childbirth, particularly in the most vulnerable populations with the least access to quality emergency obstetric care. The use of the device could also contribute to efforts to counteract the increasing trend in the use of caesarean sections observed in many countries. Further studies should examine if the device contributes to a decrease in perinatal infections acquired through the birth canal.

### Ethical issues

Often new devices and procedures are introduced in medical practice without having been properly evaluated in controlled experiments with patients [19] and/or sufficient laboratory testing [20]. One ethical pitfall of this approach is that it does not offer adequate protection to the patients receiving the new treatment nor does it provide sufficient evidence on safety and efficacy in order for health care providers to evaluate appropriateness of its use [19,20]. The present study aims at studying safety and feasibility of a new device by means of a thoroughly reviewed study protocol and with appropriate informed consent procedures. These steps will ensure that the participating women and their unborn children are not exposed to uncontrolled testing and use of a device. Avoiding this kind of uncontrolled experimentation is one of the major reasons for enrolling pregnant women in rigorous and controlled biomedical research [21].

In the context of phase 1 of the proposed study, women enrolled in the trial will be volunteers who are experiencing a normal pregnancy and are anticipating physiological labour and delivery. This is not therapeutic research and there is no significant direct benefit to the participating women and their babies (although it is expected that the duration of the 2nd stage of labour will be reduced, which might result in less pain and less pushing efforts). The benefits resulting from this study (if the device proves efficacious) will apply to all pregnant women worldwide. A safe, easy to apply, inexpensive device that can be used to assist vaginal birth has the potential to significantly impact maternal and perinatal morbidity and even mortality. In phase 2, women with prolonged second stage of labour might experience direct benefits.

We believe that the use of the Odón device during normal labour does not pose any additional risk to the mother or her fetus than unassisted labour and delivery. We therefore consider the study risks to be minimal. However we cannot exclude unknown risks. Determining potential risks associated with the use of the device is one of the major objectives of the proposed study.

To ensure that potential research participants are fully informed of the foreseeable risks and benefits of study participation, and are aware that they are able to choose to participate or withdraw from the study at any time, these steps will be followed:

•Potential participants will be informed about the study by a midwife/nurse to avoid any intimidation that might be caused if the attending obstetrician approached them about the study;

•Potential participants will have the opportunity to take the information sheet home and discuss it with the partner and family, and can make a decision about study participation when she comes back for the next antenatal care visit;

•Potential participants will be guaranteed easy access to the attending obstetrician and other study personnel in case any questions or concerns.

### Data and Safety Monitoring Board (DSMB)

A DSMB will be established to ensure independent and regular monitoring of the study, and evaluation of results including adverse events. The role of the DSMB will be:

•to safeguard the interests of study‘s participants, potential participants, investigators and sponsors

•to assess the safety and feasibility of the Odón device according to data available at a predefined schedule

•to monitor the study‘s overall conduct and quality, and protect its validity and credibility

•to make recommendations to the study Steering Committee concerning continuation/termination of study or any other modification necessary based on the observed effects of the intervention.

Detailed description of the roles and responsibilities of the DSMC and operationalization in this study are contained in the DSMB charter.

## Ethical approvals

This protocol has been approved by the Scientific and Ethical Review Group of the UNDP/UNFPA/UNICEF/WHO/World Bank Special Programme of Research, Development and Research Training in Human Reproduction at the Department of Reproductive Health and Research of WHO, and the WHO Research Ethics Review Committee, Geneva, Switzerland. The ethics committee of each participating institution and the National Drug Regulatory Agency of Argentina also approved the study.

## Competing interest

KJ is an employee of BD and provided technical advice on issues related to the potential industrial production of the device. BD has a temporary exclusivity agreement with the inventor for the licensing of the device. All other authors listed declared that they have no competing interest.

## Authors’ contribution

JS, HK and MM conceived the study and developed the study protocol. All authors participated in the design of the study and contributed to the writing and revising of the study protocol and of this manuscript. All authors read and approved the final manuscript.
